# Rediscovering Olive Mill Wastewater: New Chemical Insights Through Untargeted UHPLC-QTOF-MS Data-Dependent Analysis Approach

**DOI:** 10.3390/foods14234128

**Published:** 2025-12-02

**Authors:** Laura Alessandroni, Massimo Ricciutelli, Simone Angeloni, Giovanni Caprioli, Gianni Sagratini

**Affiliations:** Chemistry Interdisciplinary Project (ChIP), School of Pharmacy, University of Camerino, 62032 Camerino, Italy; laura.alessandroni@unicam.it (L.A.); massimo.ricciutelli@unicam.it (M.R.); giovanni.caprioli@unicam.it (G.C.); gianni.sagratini@unicam.it (G.S.)

**Keywords:** olive mill wastewater, bioactive compounds, metabolites, olive oil by-products, olive oil, LC-MS, untargeted analysis

## Abstract

With the advent of new analytical technologies and the urgent environmental problem, reopening investigations into polluting waste matrices becomes a priority. Olive mill wastewater is a pollutant and phytotoxic by-product of olive oil production. An untargeted UHPLC-QTOF analysis of three olive mill wastewaters from three different olive cultivars was performed, and modern informatic platforms were involved to characterize the chemical components in-depth. Data elaboration and statistical analysis confirmed the differences between samples and revealed a total of 364 annotated compounds, including iridoids, phenolic compounds, flavonoids, lignans, cinnamic acid derivatives, and pyrrolidine derivatives. Many of these metabolites, including compounds with known antioxidant and bioactive potential, are scarcely reported in olive products and by-products. The outcomes of this work could be useful for rethinking olive mill wastewater as a source of bioactive compounds to develop and optimize new detoxification strategies.

## 1. Introduction

Worldwide, over 10 million and 800 thousand hectares are devoted to olive cultivation; 97% of this land is concentrated in the Mediterranean region, where the olive tree (*Olea europaea* L.) has long played a significant role in economy, diet, and habits. Among the European nations, Italy is the second producer, accounting for 33% of EU output, after Spain, which, with over 826 thousand tons each year, is the main player worldwide [1]. *O. europaea* is mainly cultivated to produce olive oil, one of the oldest and healthier condiments. The precious and most appreciated virgin olive oil is obtained exclusively with mechanical extraction techniques (i.e., cold pressurization) from ripe fruit, in the period between September and November (EU Regulation No 1308/2013). From a chemical standpoint, olive oil has been extensively examined due to its advantageous fatty acid profile; moreover, recent studies have focused on identification and quantification of minor components, such as phenolic substances, to understand their bioactivity [2,3,4]. However, the olive oil production chain leads to high amount of waste and by-products in short periods of time, being the base of a severe environmental issue in the Mediterranean areas. Harvesting waste is represented by olive leaves and wood, while the solid and liquid extraction by-products are olive pomace (OP) and olive mill wastewater (OMWW), respectively. Olive pomace is a mixture of olive pulp and stones, which represents 50−60% of the total olive mass. OP is often reused in different fields, such as soil fertilizers, a biomaterial construction base, fuel biomass, and a squalene-rich matrix in cosmetics, while OMWW is known for its phytotoxic effects related to the high phenolic content [5]. Depending on the extraction method, the milling process generates approximately 0.5–1.5 m^3^ of wastewater per ton of olives, and 98% of this, corresponding to 30 million m^3^/year, is produced in the Mediterranean basin [6]. OMWW is constituted by 83−94% *w*/*w* of water and 4−16% *w*/*w* of organic fraction, while inorganic compounds and minerals account for 0.4−2.5% *w*/*w* [7]. According to the scientific literature, several research works have been performed to test and optimize purification strategies, including biological, chemical, and physical treatments [6,8,9,10]. Nowadays, more than 50 phenolic secondary metabolites have been already detected and quantified in OMWW [11].

Despite this, recently, classical methodologies have been replaced by advanced analytical strategies, in which MS-based techniques play a crucial role. In particular, the introduction of high-resolution mass spectrometers together with modern informatic platforms has paved the way for the application of untargeted analytical methods, which could lead to the identification of new molecules in food and processing by-products. In this line, the “foodomics” area was introduced as a sub-field of food science to study food matrices through the application of advanced “omics” methods [12,13].

Given its rich content of phenolic compounds, secoiridoids, lignans, and other bioactive metabolites, OMWW represents a promising yet underexploited source for potential applications in food, nutraceutical, pharmaceutical, and cosmetic technologies. A deeper chemical characterization is therefore essential to support its sustainable valorization. All these aspects contributed to the development of this research, as part of a comprehensive study about the nature and functionality of OMWW.

To the best of the authors’ knowledge, this is the first OMWW analysis with a strategy of high-resolution mass spectrometry combined with modern data elaboration generally adopted in metabolomics. Therefore, the present paper aims to automatically annotate the greatest number of polar compounds of three OMWWs from different cultivars through an untargeted UHPLC-QTOF data-dependent analysis. This modern approach allows for a broad screening of OMWW metabolites, including several molecules not commonly described in olive products. This kind of paper could provide new insights into olive oil control and research in terms of reuse and valorization of this plant-harmful matrix and new understandings to develop innovative purification and detoxification strategies.

## 2. Materials and Methods

### 2.1. Chemical and Reagents

Methanol (MeOH), propan-2-ol (isopropanol, *i*PrOH), and formic acid (99%) were LC-MS-grade and were purchased from CARLO ERBA Reagents (Milano, Italy). Ultrapure water was obtained by purified deionized water using a Milli-Q Reagent Water System (Bedford, MA, USA). *n*-Hexane was obtained from CARLO ERBA Reagents (Milano, Italy). Phenex™ RC 4 mm 0.2 μm syringe filters were bought from Phenomenex (Bologna, Italy), while CPS PTFE 25 mm 0.45 μm syringe filters were obtained from CPS Analitica (Milano, Italy). Reference and tuning mix solutions for the QTOF mass spectrometer were provided by Agilent Technologies (Santa Clara, CA, USA).

### 2.2. Samples and Samples Preparation

Olive oil mill wastewater (OMWW) was collected in October–November 2023 in a local olive mill in the Marche region, Frantoio Torresi (Potenza Picena, MC, Italy). Three different samples of OMWW were collected from the production of extra virgin olive oil (EVOO): *Sargano di Fermo* (SF) cultivar, *Peranzana* (PER) cultivar, and multivarietal (MV), respectively. The PER cultivar originates from Apulian region, while SF and MV come from Marche region (Italy). Immediately after the collection of about 2 L of each, samples were split into multiple aliquots and kept at −80 °C until use.

Three samples of each cultivar were thawed at room temperature, and a 10 mL aliquot was centrifuged for 5000 rpm for 30 min to remove solid residues. Then, 1.5 mL of the liquid fraction was further centrifuged for 1 h at 10,000 rpm, and the supernatant was collected. The lipophilic components were removed by a defatting process through liquid–liquid extraction using hexane as a solvent (0.5 mL, three times). A total of 800 µL of defatted sample was diluted with 200 µL of MeOH and mixed for 1 min. Before injection, a 2-step filtration was involved using filters with 0.45 µm and 0.22 µm pores. The samples were preliminarily tested using an HPLC 1260 Infiniti II coupled with a diode array detector (DAD) and a single-quadrupole mass spectrometer from Agilent Technologies (Santa Clara, CA, USA). For this preparatory screening with a low-resolution mass spectrometer and DAD, chromatographic separation was achieved, as reported by Ricciutelli, Marconi, Boarelli, Caprioli, Sagratini, Ballini, and Fiorini [3].

### 2.3. UHPLC-QTOF Untargeted Acquisition

The instrument adopted for the present investigation was an UHPLC 1290 Infinity II coupled with a Q-TOF 6545 from Agilent Technologies (Santa Clara, CA, USA). The chromatographic method was optimized starting from the method previously reported by Ricciutelli et al. (2017) [3]. The analytical column used for metabolite separation was a Phenomenex Synergi Polar-RP 80 Å (150 × 2 mm; 4 µm) preceded by SecurityGuard ULTRA Cartridge UHPLC Fully Porous Polar C18 2.1 mm ID column. The column was kept at 35 °C, and the injection volume was 1 µL. The mobile phase consisted of water with 0.1% formic acid (A) and MeOH:*i*PrOH (90:10, *v*/*v*) with 0.1% formic acid (B). The analyte separation was achieved at a flow rate of 0.3 mL/min, and the elution was performed in gradient mode as follows: 0−15 min, 20−60% B; 15−20 min, 60−95% B; 20−25 min, constant at 95% B; 25−26 min, 95−20% B; and 26−33 min, constant at 20%. The quality control (QC) sample was prepared by combining 300 µL of each sample. QC and blank samples were injected at the beginning of the sequence, every five injections, and at the conclusion of the sequence. A Dual Jet Stream electrospray (ESI) ionization source was installed in the high-resolution mass spectrometer, and, for mass correction, a reference solution was continuously nebulized in the ionization source during acquisition. The following parameters were set in the ionization source: gas temperature, 250 °C; drying gas, 11 L/min; nebulizer, 60 psi; sheath gas temperature, 350 °C; and sheath gas flow, 12 L/min. Capillary, nozzle, and fragmentor voltages were kept at 3500, 1000, and 110 V, respectively.

The acquisition was performed in SCAN and Auto MS/MS (data dependent analysis, DDA) modes [14]. In SCAN mode, the acquisition range was set from 50 to 1700 *m/z*, and the acquisition rate was 1 spectra/s. In Auto MS/MS mode, the rate was 1 and 3 spectra/s for MS and MS/MS acquisition, respectively, while the acquisition range was 50−1700 *m/z* for both MS and MS/MS. The precursor ions, during the MS/MS experiments, were selected based on their charge state and then their abundance. The collision energy was fixed at 30 eV. For each cycle, a maximum of four precursor ions were selected for sequential MS/MS fragmentation, while after recording three MS/MS spectra, the exclusion of given precursor ions was activated. Selection of the same ion was reactivated after 0.1 min. The DDA acquisition algorithm was applied in the iterative mode for five consecutive injections. Thus, each sample was injected five times, and within the same time window, the precursor ions selected for fragmentation in the previous injection/injections were excluded in the consecutive ones. The auto MS/MS in iterative mode was performed in duplicate. The MassHunter software package (ver. 10.0) (Agilent Technologies) was used to control the acquisition parameters of the UHPLC-QTOF instrument.

### 2.4. Data Elaboration

Agilent MassHunter Qualitative Analysis (ver. 10.0) and MS-DIAL (ver. 4.9.221218) were involved to manage, elaborate, and process the acquired data. The raw data (.d files) were converted into a suitable format (.abf) for MS-DIAL, which was used for data processing. In detail, this software automatically enabled performing peak-picking (features), alignment, integration, and annotation. Features were searched in the mass range from 50−1700 *m/z* with a minimum peak height of 1000 cps. The mass tolerance was 0.01 and 0.025 Da for MS and MS/MS, respectively, while the annotation was achieved by matching with the NIST 2020 high-resolution MS/MS library. MS-DIAL automatically performs feature annotation and calculates a total identification score based on mass accuracy, isotopic pattern, and MS/MS spectral matching. The total identification score cutoff was set at 60%. Gap filling was performed using the peak finder algorithm. After data processing, one data matrix for positive and one for negative were exported, handled, and used for statistical analysis.

### 2.5. Statistical Data Analysis

Data matrixes containing the area percentages of more than 5000 features from the three OMWWs were filtered by standard deviation to have a total of 5000 features; then, data were normalized by median and Pareto-scaled before one-way analysis of variance (ANOVA), principal component analysis (PCA), and heatmap and hierarchical analysis, which were carried out by MetaboAnalyst 6.0 [15]. After annotation, the resulting compound abundances were normalized with the Total Ion Chromatogram (TIC) areas. A positive TIC area was used for compounds annotated in the positive acquisition analysis and a negative TIC area for the ones in the negative ionization mode. TIC-normalized databases were combined to be log-transformed, Pareto-scaled, and analyzed through partial least squares–discriminant analysis (PLS-DA) to assess the discriminating annotated compounds among samples. Features with more than 50% of missing values were removed, and the remaining missing values were estimated and replaced with 1/5 of the minimum positive of the series.

## 3. Results and Discussion

The obtained spectra from the three OMWW samples revealed a complex mixture of molecules that were tentatively identified using the appropriate software (MS-DIAL) and library (NIST 2020 high-resolution MS/MS). In this section, the results are presented and discussed according to the data elaboration steps.

### 3.1. Unknown Features: PCA and Heatmap

**Figure 1:**
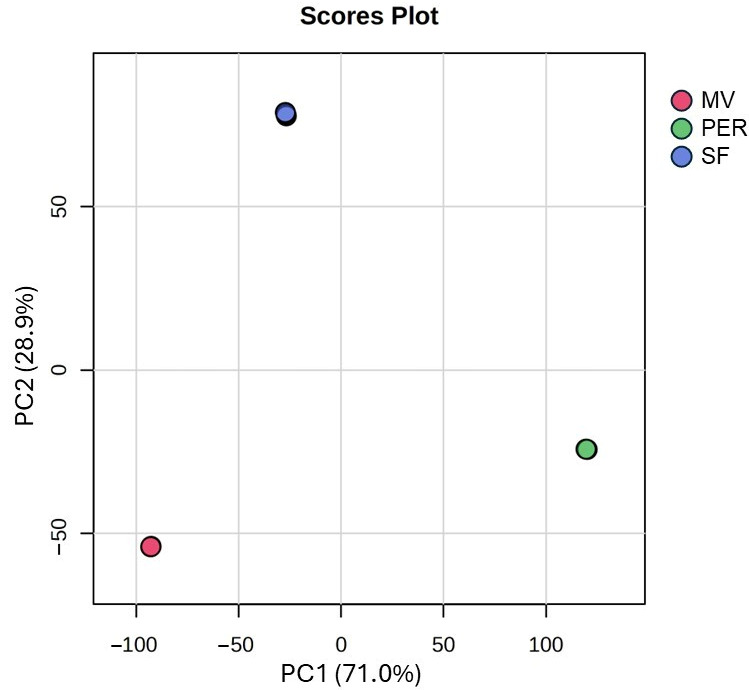
Principal components analysis scores plot of the 5000 most significant features from both polarities (PER: Peranzana; MV: multivarietal; SF: Sargano di Fermo).

**Figure 2:**
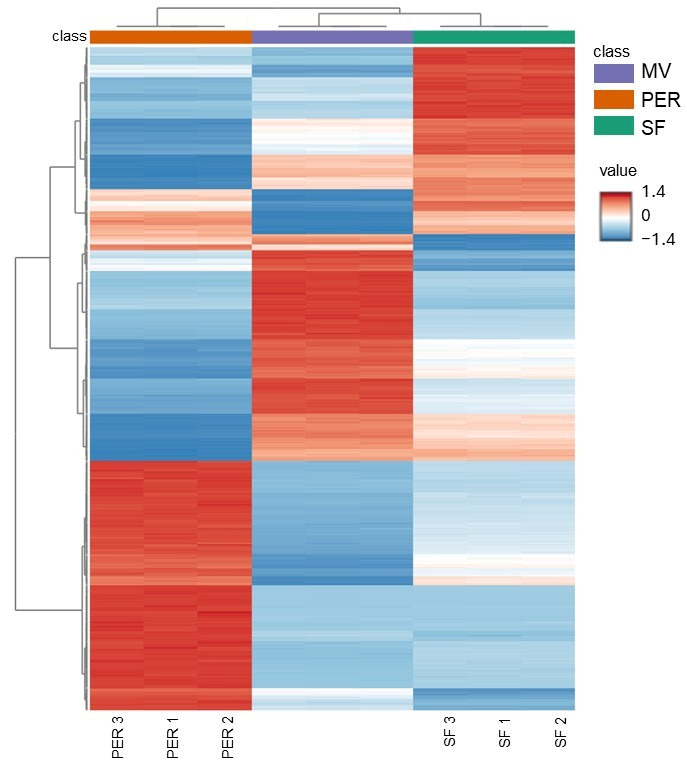
Heatmap with hierarchical clustering analysis of the most significant features for both polarities (PER: Peranzana; MV: multivarietal; SF: Sargano di Fermo; numbers represent replicates).

A total of 27,041 and 21,755 aligned features resulted from data elaboration of positive and negative acquisitions, respectively. These huge data matrices containing the area percentages were filtered by standard deviation among replicates to obtain a total of 5000 features for positive and 5000 for negative polarity. Among them, 4713 features in positive and 4703 in negative were statistically significant (one-way ANOVA, *p*-value FDR cutoff of 0.05) among the three OMWW samples. The 2500 features with a lower *p*-value from each polarity database were selected and combined in a data matrix containing 5000 features, which was analyzed and visualized with a PCA and a heatmap with hierarchical clustering. From the 2D scores plot of PCA in Figure 1, a clear separation of sample groups (SF, PER, and MV) can be noticed. The sum of the variance explained by PC1 and PC2 was 99.9%, underlining a definitely accurate response given by the principal planes. Data visualization of feature abundances is reported in the heatmap in Figure 2. In particular, each group of samples shows its own characteristics in terms of chemical composition. This is confirmed by the three well-distinguished red areas of the heatmap. The Ward hierarchical clustering revealed an interesting outcome of this screening analysis, being similar among MV and SF (Figure 2). This could be due to their origin, as both OMWWs come from Marche region cultivars, while the PER sample is an Apulian cultivar. According to previous study, OMWW properties, similarly to EVOO ones, can vary depending on different parameters, such as olive crop yield, cultivar, ripeness level, soil type, and climatic conditions [16,17]. However, further investigations employing a high number of samples of each origin will be necessary to confirm this preliminary finding.

### 3.2. MS/MS Compound Annotation

The annotation step was performed using MS-DIAL loading the NIST 2020 high-resolution MS/MS library (as an example, MS/MS spectra of some annotated compounds are reported in Figure S1). Identification scores were calculated by MS-DIAL as described in Section 2.4. After filtering unknown features and the manual handling of the database, a total of 364 compounds were annotated, with a total identification score cutoff of 60%, reaching a confidence level of 2 [18]. Among them, 223 were annotated only in positive, 120 only in negative, and 21 both in positive and negative ionization acquisitions (Table S1). Stereoisomers, e.g., (R) and (S) or D and L, could not be discriminated by the library due to their great similarity or identical MS/MS spectra. Therefore, in the present work, the nomenclature referring to stereochemistry should not be considered formative for discriminating stereoisomers, since the one suggested by the NIST library has been kept.

**Figure 3:**
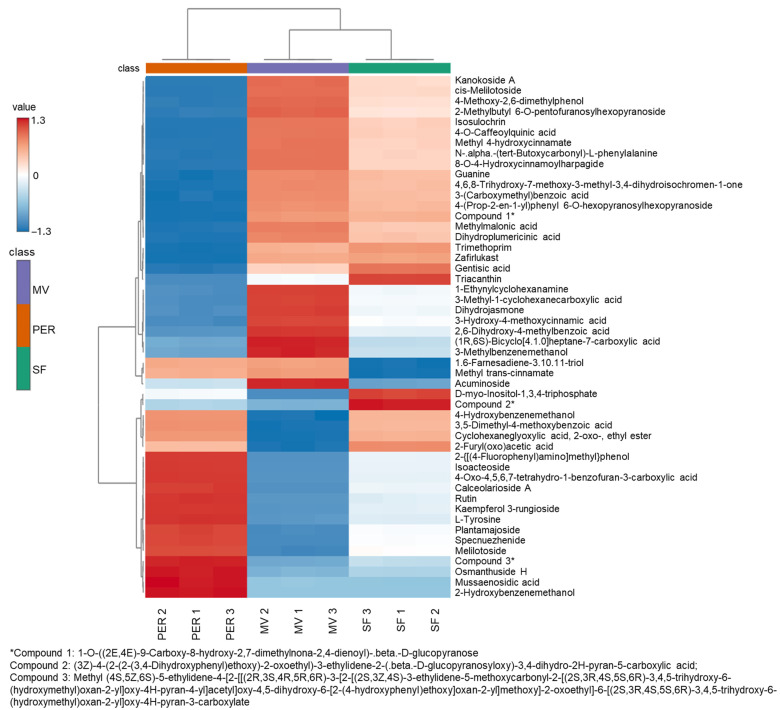
Heatmap with hierarchical clustering analysis of the top 50 ANOVA-annotated compounds (numbers after PER, MV, and SF represent replicates).

The annotated compounds with a score higher than 90% are reported in Table 1, with their retention times, experimental and reference *m/z*, adduct type, formula, InChIKey, and total score. Moreover, to better visualize the abundances of the top 50 most significant annotated compounds in the three groups of samples according to ANOVA, a heatmap with hierarchical clustering was structured (Figure 3). Each sample group showed a distinct profile containing variable levels of metabolites. This information, if studied in depth, can be useful in the control and authentication phases of olive mill products.

The top-scoring molecules were isoacteoside with 97.4% and jaslanceoside B with 97.0%. The former, also called isoverbascoside, was tentatively identified in positive and negative mode, and a total of five adduct types were registered, while the second was identified in negative mode with one adduct type. These compounds are hydroxycinnamic acid derivatives. In particular, isoverbascoside is commonly found in olive oil and olive mill wastewater [11], and, in this experiment, it was more highly concentrated in PER OMWW than in the other samples (Figure 3). During the handling process for checking the list of annotated compounds, a higher match score was obtained for forsythoside A than isoacteoside for the same feature. Although the match score was slightly higher for forsythoside A, it was decided to select the second-best scorer from the listed compounds, being isoacteoside, a molecule commonly found in this matrix. Forsythoside A presents similarities with the chosen compound, with differences just in the position of hydroxycinnamic acid as a sugar substituent; hence, some issues in the annotation procedure can occur. However, further detailed studies are necessary to clarify this aspect, in particular to understand the presence or absence of forsythoside A in olive mill wastewater, also considering its relevant positive health effects [19,20]. The structure of jaslanceoside B is an oleoside with a hydroxycinnamic acid linked in position 3 of the pyran. It was found in *Jasminum lanceolarium* and *Jasminum grandiflorum* extract, exhibiting anti-hypertensive activity [21].

To the best of the authors’ knowledge, this compound was rarely reported in olive products. The third top-scoring compound was oleoside 11-methyl ester, a hydrolytic derivative of the most common secoiridoids in olive fruit, such as oleuropein and ligstroside. Numerous chemical and enzymatic reactions, including ester hydrolysis, occur during olive oil production, and they results in metabolite structure modifications [22].

Among the best-scoring annotations in Table 1, several other glycosidic phenolic compounds are listed. 8-O-4-hydroxycinnamoylharpagide is a harpagide derivative commonly extracted from tubers of *Harpagophytum procumbens* (Burch.), also known as Devil’s Claw. Extracts of this plant, due to their concentration of harpagide and derivatives, have demonstrated valuable medicinal properties, such as anti-inflammatory, analgesic, and potential antirheumatic effects [23]. This compound was observed at a higher concentration in SF and MV OMWW than in PER, according to the heatmap in Figure 3. A content of these substances was also reported in *Melittis melissophyllum* L. agitated shoot cultures [24], but they are rarely present in olive, olive tree, and related products. Verbasoside was annotated with a score of 93.9, which is a glycoside of hydroxytyrosol. The available literature does not indicate the presence of this molecule in olive oil or olive mill wastewater, unlike its more famous derivative, verbascoside or acteoside. This molecule, which presents esterification with trans-caffeic acid at position 4 of the glucopyranosyl moiety, was largely reported as an antioxidant agent in olive mill wastewater [25,26,27] and olive leaves [28,29]. Verbascoside was annotated in negative mode, with a score of 79.6 (Table S1). Another annotated glycoside, which is also a tyrosol derivative, was salidroside. This compound was already studied as a table olive metabolite [30] and a constituent of olive-based dietary supplements [31]. Luteolin 7-glucoside, annotated in OMWW with a score of 91.5, is a luteolin-substituted glycosyloxyflavone that is largely reported in olive products [11] and renowned for its acetylcholinesterase (AChE)-inhibitory activity [29]. Secologanoside and (3-ethenyl-2-(.beta.-D-glucopyranosyloxy)-5-(methoxycarbonyl)-3,4-dihydro-2H-pyran-4-yl) acetic acid, also known as secoxyloganin, two oleoside-type secoiridoid glycosides, were annotated with a score of 93.8 and 91.8, respectively. Numerous studies have reported their abundances in the Oleaceae family, and several biological activities of oleoside-type secoiridoid glycoside-rich extracts have been reported, such as antioxidative, antitumor, anti-inflammatory, anti-diabetic, anti-obesity, neuroprotective, anti-allergic, and cardiovascular activity [32,33,34,35]. Moreover, secoxyloganin, together with 2-phenylethyl 2-O-.beta.-D-xylopyranosyl-.beta.-D-glucopyranoside, were the only molecules, together with isoacteoside, annotated in both polarities among the top-scorers listed in Table 1.

With a score of 91.4 in negative ionization mode, 4-((3R,4S)-4-hydroxy-4-(4-hydroxy-3-methoxybenzyl)-3-(hydroxymethyl)tetrahydrofuran-2-yl)-2-methoxyphenyl.beta.-D-glucopyranoside, a lignan glycoside that is structurally similar to matairesinoside, was annotated. This was extracted from *Tracheospermum asiaticum* [36,37] and from different *Centaurea* species [38], showing marked antioxidant activity. In addition, a recent study found it to be a powerful inhibitor of TMEM16A, a new target for treating lung cancer [39]. Yet, to authors knowledge, this substance has been only scarcely reported, and no data appear to be available for olive products and by-products.

The presence of cinnamic acid derivatives in olive mill wastewater is already largely described in the scientific literature [8,11]. Moreover, these compounds, together with specific secoiridoids and lignans, were proposed as olive oil authenticity biomarkers, as they are quantified in higher amounts in extra virgin olive oil than in other vegetable oils [40]. Among the cinnamic acid derivatives, sinapyl alcohol, cis-melilotoside, picroside III, 4-O-caffeoylquinic acid, melilotoside, and caffeic acid were annotated with scores of 92.0, 91.9, 91.8, 90.8, 90.0, and 90.0, respectively. According to the heatmap representation in Figure 3, melilotoside was detected in higher concentrations in OMWW from the PER cultivar, while cis-melilotoside, together with 4-O-caffeoylquinic acid, was more present in the SF and MV samples. Melilotoside and cis-melilotoside are phenolic glycosides that take their names from the botanical species from which they were isolated, i.e., *Melilotus tauricus* L. [41]. These compounds were then also found in many other plants, such as *Lavandula angustifolia* Mill. [42], *Prunus mahaleb* L. [43], or vine tea (*Ampelopsis grossedentata* Hand.-Mazz.) [44], and their anti-fatigue, anti-tyrosinase, and antimelanogenic effects were already tested. However, based on the available literature, these compounds do not appear to have been documented in olive products or by-products. 4-O-caffeoylquinic acid, or cryptochlorogenic acid, is a secondary metabolite found in high concentrations in coffee (*Coffea canephora* Pierre ex A.Froehner and *C. arabica* L.) [45] but in lower concentrations in several other plant matrices. Among iridoid glycosides, picroside III and II (Table S1) were tentatively identified. Picroside metabolites are extensively used in traditional Asian medicine for the treatment of various immune-related diseases, and they are commonly extracted from *Picrorhiza* (Scrophulariaceae) genus plants [46,47]. It has been demonstrated that extracts from this plant, which contain picroside I, II, and III, display antioxidant properties comparable to superoxide dismutase [48]. However, as far as the authors know, there is currently no published evidence on these chemicals in olive goods and by-products.

Among bioactive molecules, two pyrrolidine derivatives were annotated with a score higher than 90, namely, stachydrine and 1-pyrrolidinecarboximidamide. Stachydrine, or proline betaine, is known as the major bioactive ingredient in *Leonurus japonicus* leaves with significant pharmacological effects [49]. Remarkably, stachydrine demonstrates protective effects on the heart through various mechanisms, including reducing inflammation, fighting oxidative stress, preventing cell death, and regulating calcium levels [49,50,51]. Nevertheless, to the authors’ knowledge, published evidence of these substances in olive products or by-products is currently lacking.

### 3.3. OMWW Sample Discrimination

**Figure 4:**
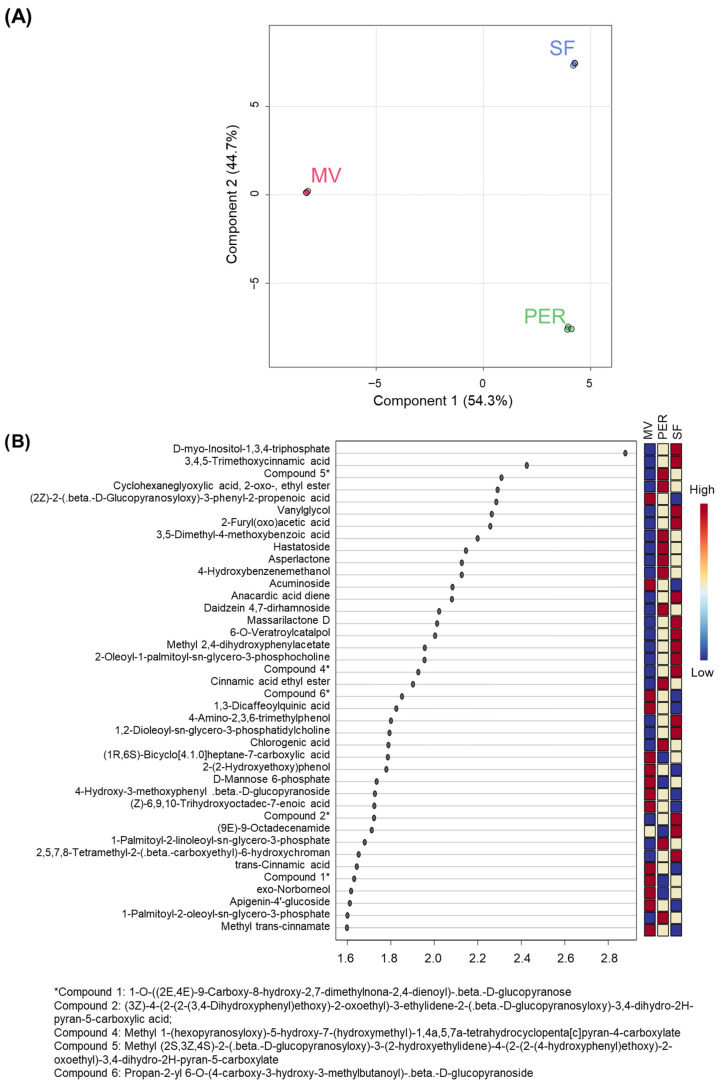
Partial least squares–discriminant analysis of the TIC-normalized database of the 364 annotated compounds. (**A**) Scores plot underlining reproducibility and discrimination; (**B**) top 40 variables with VIP scores higher than 1.5 (PER: Peranzana; MV: multivarietal; SF: Sargano di Fermo).

PLS-DA was applied to the data matrix containing the area percentages of the 364 annotated compounds with identification scores higher than 60% to better visualize the features responsible for sample discrimination. Figure 4A shows the scores plot; the top 40 annotated compounds with VIP scores higher than 1.5 are reported in Figure 4B, with the colored boxes on the right indicating the feature abundance in the sample groups. The three OMWWs were completely discriminated with high accuracy (54.3% and 44.7% for the first and second components, respectively).

The most discriminant molecules were D-myo-inositol-1,3,4-triphosphate and 3,4,5-trimethoxycinnamic acid, with VIP scores of 2.88 and 2.43, respectively. Both features were more abundant in the SF OMWW followed by the PER and MV OMWWs. The first one belongs to a large family of intracellular messengers, i.e., inositol phosphates and pyrophosphates [52]. Although the biosynthesis and exact role of these inositol derivatives are not fully comprehended in plant organisms, they might function as supporting modulators of metabolism in regulating biotic and abiotic stress responses [53]. Meanwhile, the second one, 3,4,5-trimethoxycinnamic acid, is one of the cinnamic acid derivatives synthesized and metabolized by *Clostridium* sp. In agreement with the present work, Chamkha et al. [54] isolated two new strains of *Clostridium* in OMWW after enrichment cultures on cinnamic acid; therefore, the presence of different derivatives could be related to the microbial fermentation metabolism. Indeed, other derivatives of cinnamic acids, such as cinnamic acid ethyl ester, methyl trans-cinnamate, and trans-cinnamic acid itself, emerged as important features for sample discrimination with diverse abundances in the three OMWWs.

In Figure 4B, several phenolic metabolites, such as vanylglycol, 3,5-dimethyl-4-methoxybenzoic acid, 4-hydroxybenzenemethanol, daidzein 4,7-dirhamnoside, methyl 2,4-dihydroxyphenylacetate, 1,3-dicaffeoylquinic acid, 4-amino-2,3,6-trimethylphenol, chlorogenic acid, 2-(2-hydroxyethoxy)phenol, and apigenin-4′-glucoside, are also reported, with VIP scores higher than 1.5. Besides chlorogenic acid, which has been already reported in OMWW [55], to the best of the authors’ knowledge, the other metabolites are annotated for the first time in OMWW. Sample separation, shown in Figure 4A, was also affected by some phospholipids, i.e., 2-oleoyl-1-palmitoyl-sn-glycero-3-phosphocholine, 1,2-dioleoyl-sn-glycero-3-phosphatidylcholine, 1-palmitoyl-2-linoleoyl-sn-glycero-3-phosphate, and 1-palmitoyl-2-oleoyl-sn-glycero-3-phosphate. All these molecules were more abundant in the PER and SF OMWWs and are reported for the first time in this waste product.

The clear discrimination and the high number of annotated compounds with VIP > 1 (discriminative variables) underline the great differences between the analyzed samples. Sample variability could be related to several factors, such as the cultivar, harvesting region, picking method, and other numerous parameters.

## 4. Conclusions

A deep understanding of the chemical composition of olive mill wastewater could facilitate the development of more specific detoxification techniques and introduce new perspectives in terms of reusing it as a source of bioactive compounds. In the present research, a total of 364 compounds were annotated in three OMWWs from different olive cultivars. Many of them showed documented biological activities, such as antioxidant activity, and they have rarely been documented in olive products; to confirm their presence, further targeted studies with authentic standards are necessary. Furthermore, the highlighted differences in the content of bioactive compounds, potentially related to cultivar and origin, should be further evaluated by gathering and studying a significant number of samples.

Future work will focus on applying advanced omics-based analytical approaches—such as metabolomics, lipidomics, and molecular networking—to deepen the structural characterization of OMWW metabolites and monitor their evolution during processing. These tools could lead to the development of innovative recovery and purification methods focused on selectively isolating valuable bioactive compounds. Moreover, targeted analysis will help evaluate the biological activity, health-enhancing potential, and stability of the newly identified molecules, allowing for their potential incorporation into food, nutraceutical, pharmaceutical, or cosmetic products. Finally, assessing the economic feasibility and environmental impact of OMWW valorization strategies will be essential to promote the development of sustainable, circular processes within the olive oil industry.

## Figures and Tables

**Table 1:** Annotated compounds with a score higher than 90% with their retention times (Rt), experimental and reference *m/z*, adduct type and polarity, formula, and InChIKey.

Rt (min)	Experimental *m/z*	Metabolite Name	Adduct Type	Reference *m/z*	Formula	InChIKey	Total Score ^1^
9.52	642.2386, 625.2128, 325.0931, 471.1500, 623.2021	Isoacteoside	[M+H−C_14_H_20_O_7_]^+^, [M+H−C_8_H_10_O_3_]^+^, [M+NH_4_]+, [M+H]^+^, [M−H]^−^	642.2392, 625.2127, 325.0918, 471.1497, 623.1981	C_29_H_36_O_15_	FNMHEHXNBNCPCI-QEOJJFGVSA-N	**97.4; 84.0; 74.0; 69.0; 87.3**
13.25	177.0559	Jaslanceoside B	[M−H−C_16_H_20_O_11_]^−^	177.0557	C_26_H_30_O_14_	MGEVYVDQMTWJNV-HOPHSATRSA-N	**97.0**
5.61	403.1263	Oleoside 11-methyl ester	[M−H]^−^	403.1246	C_17_H_24_O_11_	XSCVKBFEPYGZSL-JYVCFIOWSA-N	**95.3**
8.30	163.0407	8-O-4-Hydroxycinnamoylharpagide	[M−H−C_15_H_22_O_9_]^−^	163.0401	C_24_H_30_O_12_	AZKQDXZMKREFDY-LGKDJQOASA-N	**94.2**
2.67	461.1675	Verbasoside	[M−H]^−^	461.1664	C_20_H_30_O_12_	DORPKYRPJIIARM-GYAWPQPFSA-N	**93.9**
3.87	389.1140	Secologanoside	[M−H]^−^	389.1089	C_16_H_22_O_11_	RGTONEMDTVVDMY-UHFFFAOYSA-N	**93.8**
2.88	114.1029	1-Pyrrolidinecarboximidamide	[M+H]^+^	114.1026	C_5_H_11_N_3_	PIGIRWPZTWLVLB-UHFFFAOYSA-N	**93.1**
1.54	124.0393	Nicotinic acid	[M+H]^+^	124.0393	C_6_H_5_NO_2_	PVNIIMVLHYAWGP-UHFFFAOYSA-N	**92.5**
4.17	107.0495	4-Hydroxybenzenemethanol	[M+H−H_2_O]^+^	107.0491	C_7_H_8_O_2_	BVJSUAQZOZWCKN-UHFFFAOYSA-N	**92.3**
1.40	195.0533	D-Gluconic acid	[M−H]^−^	195.0510	C_6_H_12_O_7_	RGHNJXZEOKUKBD-SQOUGZDYSA-N	**92.0**
5.45	193.0863	Sinapyl alcohol	[M+H−H_2_O]^+^	193.0859	C_11_H_14_O_4_	LZFOPEXOUVTGJS-ONEGZZNKSA-N	**92.0**
8.30	119.0501	cis-Melilotoside	[M−H−C_7_H_10_O_7_]^−^	119.0502	C_15_H_18_O_8_	GVRIYIMNJGULCZ-QLFWQTQQSA-N	**91.9**
5.61	807.2576, 403.1248, 243.0867	(3-Ethenyl-2-(.β.-D-glucopyranosyloxy)-5-(methoxycarbonyl)-3,4-dihydro-2H-pyran-4-yl)acetic acid	[M+H−C_6_H_10_O_5_]^+^, [2M−H]^−^, [M−H]^−^	807.2564, 403.1246, 243.0863	C_17_H_24_O_11_	MQLSOVRLZHTATK-WNCYHATQSA-N	**91.8; 87.4; 82.3**
10.51	193.0548	Picroside III	[M−H−C_15_H_20_O_9_]^−^	193.0506	C_25_H_30_O_13_	RMSKZOXJAHOIER-GGKKSNITSA-N	**91.8**
10.95	447.0933	Luteolin 7-glucoside	[M−H]^−^	447.0933	C_21_H_20_O_11_	PEFNSGRTCBGNAN-QNDFHXLGSA-N	**91.5**
5.74	583.2034	4-((3R,4S)-4-Hydroxy-4-(4-hydroxy-3-methoxybenzyl)-3-(hydroxymethyl)tetrahydrofuran-2-yl)-2-methoxyphenyl .β.-D-glucopyranoside	[M+CHO_2_]^−^	583.2033	C_26_H_34_O_12_	OXHVZEZYYQQCRJ-ZLNFVOGTSA-N	**91.4**
3.26	345.1215	Salidroside	[M+CHO_2_]^−^	345.1191	C_14_H_20_O_7_	ILRCGYURZSFMEG-RKQHYHRCSA-N	**90.9**
3.16	329.0881, 659.1824	4-(Hexopyranosyloxy)-3-methoxybenzoic acid	[M−H]^−^, [2M−H]^−^	329.0878, 659.1829	C_14_H_18_O_9_	JYFOSWJYZIVJPO-UHFFFAOYSA-N	**90.8; 82.2**
5.80	179.0418	4-O-Caffeoylquinic acid	[M−H−C_7_H_10_O_5_]^−^	179.0350	C_16_H_18_O_9_	GYFFKZTYYAFCTR-JUHZACGLSA-N	**90.8**
1.78	295.1035	4-(.β.-D-Glucopyranosyloxy)benzyl 3-(.β.-D-glucopyranosyloxy)-2-(((2Z)-3-(4-hydroxyphenyl)prop-2-enoyl)oxy)-3-methylbutanoate	[M−H−C_22_H_22_O_8_]^−^	295.1035	C_33_H_42_O_17_	LZXXRASHAINSDN-ZLFKWPTHSA-N	**90.7**
1.52	144.1056	Stachydrine	[M+H]^+^	144.1019	C_7_H_13_NO_2_	CMUNUTVVOOHQPW-UHFFFAOYSA-N	**90.6**
3.30	197.0813	4-(2-Methyl-6-oxopyran-3-yl)butanoic acid	[M+H]^+^	197.0808	C_10_H_12_O_4_	QFJCZUUIDDALPA-UHFFFAOYSA-N	**90.5**
6.28	415.1610, 461.1748, 434.2024, 439.1574	2-Phenylethyl 2-O-.β.-D-xylopyranosyl-.β.-D-glucopyranoside	[M−CHO_2_]^−^, [M+NH_4_]^+^, [M+Na]^+^, [M−H]^−^	415.1610, 461.1665, 434.2021, 439.1575	C_19_H_28_O_10_	CGZNTHDUAFIKOH-BMVMOQKNSA-N	**90.2; 82.0; 81.4; 71.6**
5.11	171.1021	3-Cyclohexyl-3-oxopropanoic acid ethyl ester	[M+H−C_2_H_4_]^+^	171.1016	C_11_H_18_O_3_	ASYASKBLHYSMEG-UHFFFAOYSA-N	**90.0**
6.66	181.0497	Caffeic acid	[M+H]^+^	181.0495	C_9_H_8_O_4_	QAIPRVGONGVQAS-DUXPYHPUSA-N	**90.0**
4.25	651.1934, 325.0939	Melilotoside	[M−H]^−^, [2M−H]^−^	651.1931, 325.0929	C_15_H_18_O_8_	GVRIYIMNJGULCZ-ZMKUSUEASA-N	**90.0; 87.4**

^1^ Different scores are related to different adducts of the same metabolite.

## Data Availability

The original contributions presented in the study are included in the article/Supplementary Material, further inquiries can be directed to the corresponding author.

## Supplementary Materials

The following supporting information can be downloaded at: https://www.mdpi.com/article/10.3390/foods14234128/s1, Figure S1: MS/MS spectra of the three highest scored annotated compounds (isoacteoside, jaslanceoside B, oleoside 11-methyl ester) and their reference spectra of NIST 2020 MS/MS library (in red); Table S1: Total list of annotated compounds in OMWW samples.

## Abbreviations

The following abbreviations are used in this manuscript:

OMWW	Olive mill wastewater
EVOO	Extra-virgin olive oil
PER	Samples from Peranzana olive cultivar
SF	Samples from Sargano di Fermo olive cultivar
MV	Samples from multivarietal oil production
DDA	Data-dependent analysis
TIC	Total ion chromatogram
PLS-DA	Partial least squares–discriminant analysis
VIP	Variables important in projection
PCA	Principal components analysis
